# *Candida* species detection in patients with chronic periodontitis: A systematic review and meta‐analysis

**DOI:** 10.1002/cre2.635

**Published:** 2022-07-28

**Authors:** Ekaterina Slazhneva, Ekaterina Tikhomirova, Victor Tsarev, Liudmila Orekhova, Ekaterina Loboda, Victoria Atrushkevich

**Affiliations:** ^1^ Department of Periodontology A.I. Yevdokimov Moscow State University of Medicine and Dentistry Мoscow Russian Federation; ^2^ Department of Microbiology, Virology, Immunology A.I. Yevdokimow Moscow State University of Medicine and Dentistry Moscow Russian Federation; ^3^ Laboratory of Molecular Biological Research Research Medical and Dental Institute Мoscow Russian Federation; ^4^ Department of Restorative Dentistry and Periodontology First Pavlov State Medical University of St Petersburg St Petersburg Russian Federation

**Keywords:** *Candida*, chronic periodontitis, fungi, periodontal diseases

## Abstract

**Objectives:**

To assess the *Candida* species occurrence rate and concentration in periodontal pockets in chronic periodontitis (CP) by meta‐analysis.

**Materials and Methods:**

A search was performed of articles published between January 1, 2010, and October 1, 2020, in English and in Russian, in the electronic databases MEDLINE‐PubMed, Google Scholar, The Cochrane Library, ClinicalTrials.gov, Research Gate, eLIBRARY, and Cyberleninka (PROSPEROCRD42021234831). The odds ratio (OR), standardized mean difference (SMD), and 95% confidence interval (CI) were calculated using Review Manager 5.4.1 to compare the risk of CP when *Candida* spp. were detected in the gingival sulcus or periodontal pocket and to compare *Candida* spp. density counts in patients with CP and periodontally healthy patients.

**Results:**

Twenty‐six studies were included in the systematic review and 11 were included in the meta‐analysis. The results showed that *Candida* spp. may increase the chance of CP development by 1.76 times (OR = 1.76; 95% CI = 1.04–2.99; *Z* = 2.10; *p* = .04; *I* ^2^ = 61%). More *Candida* spp. were found in patients with CP than in periodontally healthy patients (SMD = 1.58; 95% CI = 0.15−3.02; *p* = .03; *I* ^2^ = 98%). No data were found relating to the statistically significant influence of *Candida glabrata, Candida krusei* and *Candida tropicalis* on CP development.

**Conclusion:**

We found that *Candida albicans* insignificantly increased the risk of CP development but, due to the heterogeneity of the included studies, further research is necessary to determine the exact role of *Candida* spp. in the development and course of the inflammatory periodontal diseases.

## 1 INTRODUCTION

Chronic periodontitis (CP) is a multifactorial inflammatory disease where periodontal pathogens, mainly *Porphyromonas gingivalis, Tannerella forsythia, Treponema denticola, Prevotella intermedia*, and *Aggregatibacter actinomycetemcomitans* play a key role in the development and progression of the disease (Colombo & Tanner, 2019; Lamont et al., 2018; Roberts & Darveau, 2015). They have high pathogenic potential and form a complex bacterial society known as the biofilm (Mira et al., 2017). Besides periodontal pathogens, dental plaque contains other bacteria as well as fungi and viruses whose role is actively studied. Many researchers consider that yeast‐like fungi, specifically *Candida* spp., are one of the important causes of the development, progression, and complicated course of CP (Canabarro et al., 2013; Razina et al., 2017; Sardi, Duque, Mariano, et al., 2012).

*Candida* spp. are opportunistic microorganisms that colonize the oral mucosa and skin of healthy people (De Oliveira et al., 2007). *Candida* spp. growth is considered to be a warning sign of an immune disorder. These opportunistic microorganisms can persist for a long time in different oral niches without clinical manifestations; however, when the immune status is lowered (e.g., in patients with diabetes mellitus or with immunodeficiencies, in young children or in the elderly), or their environment changes, their virulence factors can cause disease (Colombo et al., 2016; Gaffen & Moutsopoulos, 2020). *Candida albicans* is the most common spp. while other spp. have been isolated from the oral cavity: *Candida glabrata, Candida tropicalis, Candida parapsilosis, Candida krusei*, and *Candida dubliniensis* (Sardi et al., 2010).

The high compliance of *C. albicans* allows it to colonize in different media creating mixed biofilms with commensal as well as pathogenic bacteria in aerobic and anaerobic conditions (Thein et al., 2006). The ability of the yeast to coexist with commensal and pathogenic bacteria is an important factor in their virulence which grants both microorganisms new characteristics and allows them to colonize new niches (Bamford et al., 2009; Bartnicka et al., 2019; Bernard et al., 2020; Sultan et al., 2018; Wu et al., 2015). It is worth highlighting that *Candida* spp. can adapt in different oral niches expressing different phenotypes and virulence factors according to the pH, oxygen, or polysaccharide availability. The periodontal pocket and gingival crevicular fluid are favorable media for *C. albicans* germination and hyphal tip growth. In comparison with yeast cells, *Candida* hyphae are more able to attach to host cells and penetrate the tissues (Bartnicka et al., 2019). *C. аlbicans* can interact with *Streptococcus gordonii*, *Fusobacterium nucleatum*, *P. gingivalis*, and *A. actinomycetemcomitans*, to form mixed biofilms, which makes *C. аlbicans* an active participant in the inflammatory‐destructive process in periodontal diseases (Bartnicka et al., 2019; Kornman, 2005).

The interaction between *C. albicans* and microorganisms seems to be complex and has been incompletely studied while the role of *C. albicans* in the CP pathogenesis is also complex. They secrete proteinases that release toxic or antigenic agents which in turn can increase tissue inflammation and activate an immune response similar to that of *P. gingivalis* proteases (Sardi et al., 2010). *Candida* spp. are also able to secrete phospholipases which facilitate its adhesion to the tissues and destroy cellular membranes thereby promoting cytolysis (De‐La‐Torre et al., 2018).

Scaling and root planning is a gold standard in CP treatment. However, in some patients, periodontitis is resistant to conventional treatment and requires systemic antibiotic therapy. This may promote active yeast growth which adversely affects the course of the CP (De‐La‐Torre et al., 2018; Sardi, Duque et al., 2011). Thus, refractory periodontitis may be linked to uncontrolled growth and replacement by the yeast‐like fungi in a periodontal pocket.

A systematic analysis of the literature and meta‐analysis of data to compare the detection rate and density counts of *Candida* spp in patients with CP and with periodontally healthy patients can explain their role in the origin and development of CP.

In this review, we wanted to look in detail at the occurrence rate of yeast‐like fungi in CP and assess their density counts in periodontal pockets by analyzing the clinical studies published over the last 10 years.

## 2 MATERIALS AND METHODS

### 2.1 Review questions

A systematic review and meta‐analysis was performed to compare the occurrence rate of *Candida* spp. in a periodontal pocket or a gingival sulcus in patients with CP and periodontally healthy patients, to determine the potential role of the yeast‐like fungi in the development and course of CP.

### 2.2 Search strategy, study selection, and data extraction

This systematic review and meta‐analysis was registered on PROSPERO (International Prospective Register of Systematic Reviews), registration number CRD42021234831.

#### 2.2.1 Literature search

The criteria of the Preferred Reporting Items for Systematic Reviews and Meta‐analyses (PRISMA) (2015) were used for the systematic review and meta‐analysis (Moher et al., 2015). A detailed systematic literature search was performed of articles, published in English and Russian between January 1, 2010, to October 1, 2020, in seven electronic databases: MEDLINE‐PubMed, Google Scholar, Cochrane Library central, Clinical trial, Research Gate, eLIBRARY, CyberLeninka. The following keywords were used to perform the search: ((Candida), (Candida biofilm), (fungi),(yeast)) AND (periodontitis), (Candida) AND (periodontal disease), (Candida) AND (periodontal pocket), (oral fungal‐bacterial biofilm), (Candida‐associated periodontitis). We activated the filter “human” in the PubMed database. Reference bibliographic lists of the retrieved found publications for full‐text screening were reviewed and potentially significant studies were manually selected. No language, country, observation duration, and subject characteristics (race, age, and sex) were introduced.

#### 2.2.2 Study selection

The search results were downloaded to a bibliographic database (Mendeley Reference Manager) to facilitate duplicate removal and cross‐reference checks.

Two authors (E. S., E. T.) independently screened the titles and abstracts of the entries identified in the search of all articles against the below pre‐defined inclusion and exclusion criteria.

Inclusion criteria were as follows:

- randomized and nonrandomized clinical studies, observational studies (cross‐sectional, case−control);
- patients over 18 years old, independent of race, nationality, sex;
- patients with CP without periodontal treatment, antibiotic, and antifungal therapy in the previous 6 months;
- *Candida* spp. isolation from the content of a periodontal pocket, subgingival biofilm, subgingival dental plaque, gingival sulcus;
- determination of *Candida* spp. detection rate;
- *Candida* spp. density counts in periodontal pockets;
- full‐text article availability;
- English and Russian‐language publications.

Exclusion criteria were as follows:

- reviews, meta‐analyses, clinical case studies, and reports;
- in vitro studies;
- studies with incomplete initial data;
- studies where only the abstract was available;
- studies where *Candida* spp. were detected in the saliva and on the oral mucosa;
- studies on oral candidiasis;
- the articles reviewed another pathology but not chronic generalized periodontitis;
- patient was a child (under 18 years old);
- the study included only subjects with comorbidity and immunodeficiency conditions.

Next, the full‐text version of all studies that potentially met the eligibility criteria or for which there was insufficient information in the title and abstract to make a decision were obtained.

Any disagreements between reviewers were solved by a joint discussion.

#### 2.2.3 Data extraction

Every author independently analyzed the publications and extracted data which was entered into the predetermined information table. Any disagreements were solved by a joint discussion. The following data were recorded from all the selected studies: name of the first author, publication year, country of the study, study design, studied population characteristics, sample size, number of subjects in the studied and control groups, the technique for sampling collection from the periodontal pocket or gingival sulcus, *Candida* spp. identification technique, study result expressed in *Candida* spp. detection rate or density count.

Control and experimental groups were respectively defined as a group of subjects without signs of periodontal inflammation and a group of patients with CP independent of the disease severity. As per the studies where groups were formed according to the severity of CP, the study subjects were combined in one group. In the assessment of the *Candida* spp. density count, the mean was calculated for groups with CP of different severity, when necessary, and a standard deviation parameter was calculated according to the average standard error value.

### 2.3 Evaluation of the methodological quality of the studies

Two authors independently evaluated the quality and evidence level of every study using the Russian‐language version of the Newcastle−Ottawa scale questionnaire to assess the risk of systemic bias in nonrandomized comparative studies (Rebrova & Fediaeva, 2016).

### 2.4 Statistical analysis

Statistical analysis of the results was performed using Review Manager (RevMan) 5.4.1 software. An odds ratio (OR) and 95% confidence interval (CI) were calculated for dichotomic (binary) data while the standardized mean difference (SMD) and 95% CI were calculated for continuous data. Studies, where the above‐mentioned data could not be calculated, were not included in the statistical analysis. Results of single studies and summarized data were presented in a forest plot. The heterogeneity of the studies was assessed by *χ* ^2^ and *I* ^2^. The random effects model was used when *p* < .10 or *I* ^2^ > 50% determined significant heterogeneity across all articles. Otherwise, the fixed effects model was used. The analysis of sensitivity to the combined result stability was performed to examine the source of heterogeneity. The *Candida* spp. detection rate and individual *Candida* spp. detection rate were analyzed in groups of patients with clinically healthy periodontium and CP.

### 2.5 Assessment of publication bias

The publication bias was assessed by funnel plots.

## 3 RESULTS

### 3.1 Study selection

**Figure 1:**
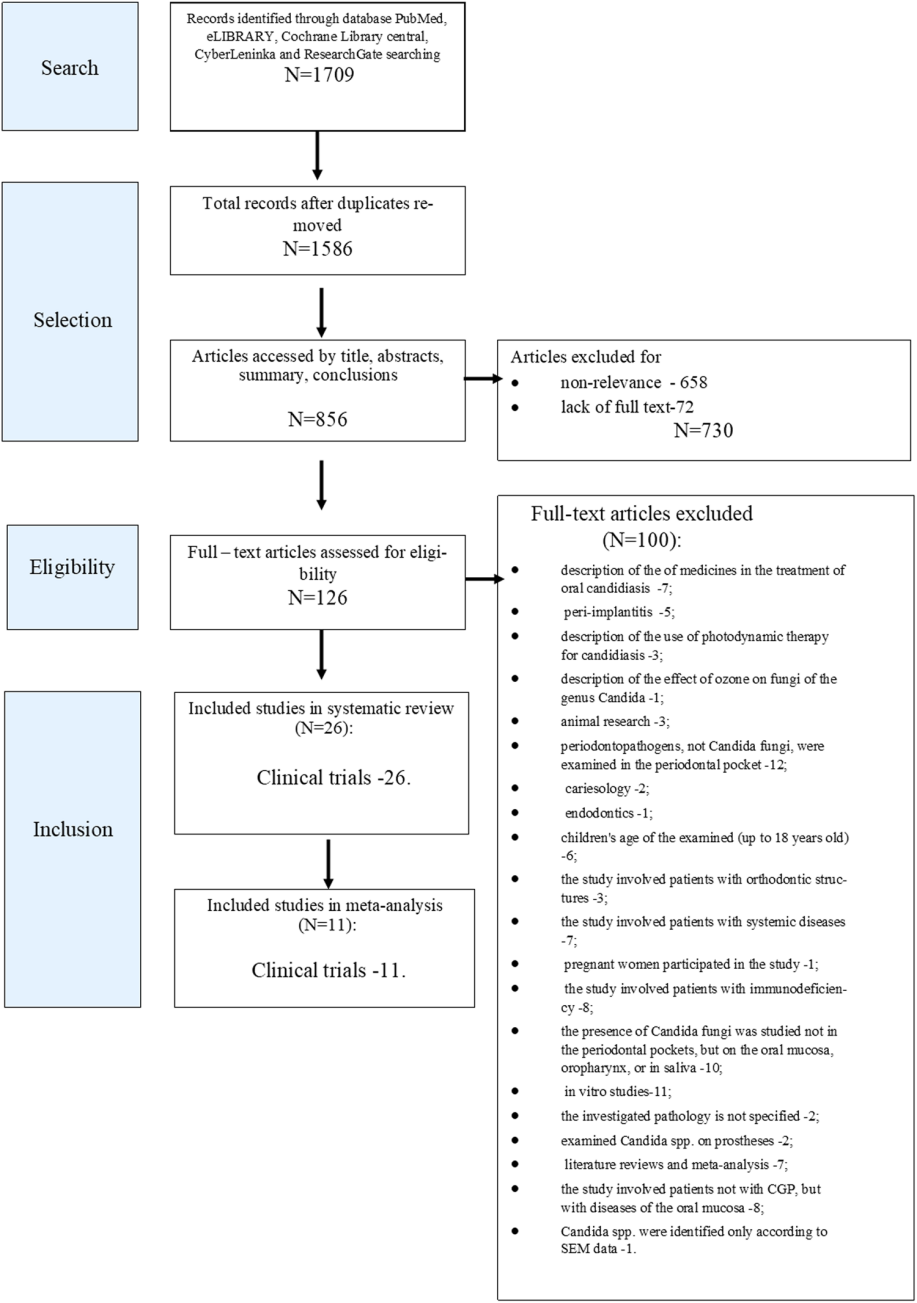
Flow diagram of study selection

The article selection process is demonstrated in Figure 1. A total of 1709 publications were initially selected by name, abstract, and publication date. One hundred and twenty‐three studies were excluded as duplicates. Six hundred and fifty‐eight more articles were excluded for nonconformance of the title and abstract to the inclusion criteria, and 72 were excluded for the absence of the full text. Sixty‐eight out of the remaining 126 full‐text articles were excluded for nonconformance to the article selection criteria. Disagreements regarding inclusion or exclusion were settled by discussion. Finally, 26 publications were selected for the systematic review by the selection criteria. Detailed analysis of the presented data revealed that 14 articles were without a control group of patients with a clinically healthy periodontium which did not allow a calculation of the OR for the meta‐analysis. Thus, the meta‐analysis included 11 articles.

### 3.2 Assessment of the methodological quality of the studies included in the meta‐analysis

An assessment of the methodological quality of the studies by the Russian‐language version of the questionnaire to assess the risk of systematic bias in nonrandomized comparative studies (Newcastle−Ottawa scale) showed that two studies (Brusca et al., 2014; Risovannaya & Lalieva, 2019) had a high risk of systematic bias—5 points on the scale, 9 (Canabarro et al., 2013; De‐La‐Torre et al., 2018; Komleva et al., 2010; Krishnan et al., 2020; Matić Petrović et al., 2015, 2019; Melekhov et al., 2013; Colombo et al., 2016; Volchenkova et al., 2017) studies had an average risk of systematic bias—6−7 points on the scale.

### 3.3 Included study characteristics

Table 1 shows the data summary of the studies included in the systematic review and meta‐analysis.

**Table 1:** Summary of the studies included in the systematic review and meta‐analysis

No.	Author, year, country	Study design	Studied population	Number of patients/subjects	Technique of sample collection from the periodontal pocket/gingival sulcus	Identification method	Result	Comments
1	**Gomes et al., 2017, Brazil** (Gomes et al., 2017)	Case−control	Male and female patients, aged 39−70 years.	15 patients with CP	Paper points	Culture, PCR	*С. albicans* was detected in	No control group (without CP and DM).
							3 (20%) patients with CP	
2	**Matić Petrović et al., 2015, Serbia** a (Matić Petrović et al., 2015)	Cross‐sectional	Male and female patients without systemic diseases with CP (aged 47.07 ± 10.869) and periodontally healthy patients (aged 43.57 ± 3.389 years).	65 subjects 30—patients with CP, 35—periodontally healthy patients	Paper points, curette (samples combined)	Culture	*Candida* spp. were detected in	Included in the meta‐analysis.
							8 (26.7%) patients with CP 9 (25.7%) periodontally healthy patients	
3	**Sardi et al., 2011, Brazil** (Sardi, Almeida et al., 2011)	Cross‐sectional	CP patients with Type 2 DM (47.1 ± 13.01 years) and without DM (45.6 ± 7.64 years) aged 31−68 years.	10 patients with CP (10 patients with CP and and normal glucose level	Curette	PCR	*Candida* spp. were detected in	No control periodontally healthy group without DM.
							7 (70%) patients with CP	
							*C*. a*lbicans* was detected in	
							4 (40%) patients with CP	
							*C. dubliniensis* was detected in	
							4 (40%) patients with CP	
							*C. glabrata* was not detected	
							*C. tropicalis* was not detected	
4	**Rams et al., 2014, USA** a (Rams et al., 2014)	Cross‐sectional	Male and female patients with CP, without systemic diseases, aged 35−78 years (50.5 ± 9.9).	400 patients.	Paper points	Culture	*Candida* spp. were not detected	No control group (without CP and DM).
5	**Razina et al., 2017, Russia** (Razina et al., 2017)	Cross‐sectional	Male and female patients aged 27−62 years (53.98 ± 1.06) with CP without systemic diseases.	90 patients with CP	Paper points	Culture	*Candida* spp. were detected in	No control group with periodontally healthy patients.
							42 (46.7%) patients with CP. *Candida* spp. density was 1.5 ± 1.9 lg CFU/ml patients with CP	
6	**De‐La‐Torre et al., 2018, Spain** a (De‐La‐Torre et al., 2018)	Cross‐sectional	Male and female patients without systemic diseases aged 30−81 years (mean age 48.2years).	155 subjects 66 patients with CP 89 periodontally healthy patients	Paper points	Culture, PCR	*Candida* spp. were detected in	Included in the meta‐analysis.
							24 (36.4%) patients with CP 32 (36%) periodontally healthy patients	
							*Candida* spp. density was	
							195.22 ± 557.79 CFU/ml in patients with CP 148.5 ± 552.9 CFU/ml in periodontally healthy patients	
7	**Canabaro et al., 2013, Brazil** a (Canabarro et al., 2013)	Case−control	Male and female patients aged 21−72 years.	60 subjects 40 patients with CP 20 periodontally healthy patients	Paper points	Culture	*Candida* spp. were detected in	Included in the meta‐analysis.
							12 (30%) patients with CP 3 (15%) periodontally healthy patients	
8	**Sanz‐Sánchez et al., 2015, Spain** (Sanz‐Sánchez et al., 2016)	Randomized clinical study	Male and female patients aged 25−80 years (mean age 52.8) with CP, without systemic diseases.	30 patients	Paper points	Culture	*Candida* spp. were detected in 3 (10%) patients with CP.	No control periodontally healthy group.
9a	**McManus et al., 2012, Ireland** (McManus et al., 2012)	Cross‐sectional	Male and female patients aged 26−73 years without systemic diseases.	71 subjects 21 patients with CP 50 periodontally healthy patients	Paper points	Culture, PCR	*Сandida* spp. were detected in	Control samples were obtained from the saliva of periodontally healthy subjects.
							8 (38.1%) patients with CP 16 (32%) periodontally healthy patients	
							*C. albicans* was detected in	
							8 (38.1%). patients with CP 16 (32%) periodontally healthy patients	
							*C*. *glabrata* was detected in	
							0 patients with CP 1 (2%) periodontally healthy patient	
							*C*. *dubliniensis* was detected in	
							2 (9,5%) patients with CP 0 periodontally healthy patients	
							*C. kefyr* was not detected.	
							*C. parapsilosis* was detected in	
							1 (4.5%) patients with CP 0 periodontally healthy patients	
							*Candida* spp. density was	
							3910 ± 5466 CFU/ml in patients with CP 1536 ± 2384 CFU/ml in periodontally healthy patients	
9b	**McManus et al., 2012, Ireland** (McManus et al., 2012)	Cross‐sectional	Male and female patients aged 26−73 years without systemic diseases.	71 subjects 21 patients with CP 50 periodontally healthy patients	Curette	Culture, PCR	*Сandida* spp. were detected in	Control samples were obtained from the saliva of periodontally healthy subjects.
							8 (38.1%) patients with CP 16 (32%) periodontally healthy patients	
							*C. albicans* was detected in	
							8 (38.1%). patients with CP 16 (32%) periodontally healthy patients	
							*C*. *glabrata* was detected in	
							0 patients with CP 1 (2%) periodontally healthy patient	
							*C*. *dubliniensis* was detected in	
							1 (4,8%) patients with CP 0 periodontally healthy patients	
							*C. kefyr* was detected in	
							1 (4,8%) patients with CP 0 periodontally healthy patients	
							*C. parapsilosis* was not detected	
							*Candida* spp. density was	
							3528 ± 8743 CFU/ml in patients with CP 1536 ± 2384 CFU/ml in periodontally healthy patients	
9c	**McManus et al., 2012, Ireland** (McManus et al., 2012)	Cross‐sectional	Male and female patients aged 26−73 years without systemic diseases.	71 subjects 21 patients with CP 50 periodontally healthy patients 71 patients (50 controls, 21 CP patients).	Paper points, curette (averaged result)	Culture, PCR	*Сandida* spp. were detected in 10 (47,6%) patients with CP 16 (32%) periodontally healthy patients *C. albicans* was detected in 10 (47,6%) patients with CP 16 (32%) periodontally healthy patients *C*. *glabrata* was detected in 0 patients with CP 1 (2%) periodontally healthy patient *C*. *dubliniensis* was detected in 2 (9.5%) patients with CP 0 periodontally healthy patients *C. kefyr* was detected in 1 (4.8%) patient with CP 0 periodontally healthy patients *C. parapsilosis* was detected in 1 (4,8%) patient with CP 0 periodontally healthy patients *Candida* spp. density was 3719 ± 7204 CFU/ml in patients with CP 1536 ± 2384 CFU/ml in periodontally healthy patients	Control samples were obtained from the saliva of periodontally healthy subjects.
10	**Colombo et al., 2016, Brazil** a (Colombo et al., 2016)	Cross‐sectional	Male and female patients aged 18−56 years without systemic diseases.	215 subjects 134 patients with CP 81 periodontally healthy patients	Curette	Checkerboard DNA−DNA hybridization	*Candida* spp. were detected in 74 (54%) patients with CP 28 (35%) periodontally healthy patients *Candida* spp. density count was (data taken from the diagram) 4.4Е + 05 in patients with CP 0.5Е + 05 in periodontally healthy patients	Included in the meta‐analysis.
11а	**Matic Petrovićet al., 2019, Serbia** a (Matic Petrovic et al., 2019)	Cross‐sectional	Male and female patients aged 26−73 years without systemic diseases.	78 subjects: 42 patients with CP 36 periodontally healthy patients	Paper points, curette (averaged result)	Culture	*Сandida* spp. were detected in 6 (14.3%) patients with CP 9 (25%) periodontally healthy patients	Included in the meta‐analysis.
11b	**Matic Petrovićet a**l**., 2019, Serbia ** a (Matic Petrovic et al., 2019)	Cross‐sectional	Male and female patients aged 26−73 years without systemic diseases.	78 subjects: 42 patients with CP 36 periodontally healthy patients	Paper points	Culture	*Сandida* spp. were detected in 5 (11.9%) patients with CP 6 (16.7%) periodontally healthy patients	Included in the meta‐analysis.
11с	**Matic Petrović S et al., 2019, Serbia** a (Matic Petrovic et al., 2019)	Cross‐sectional	Male and female patients aged 26−73 years without systemic diseases.	78 subjects: 42 patients with CP 36 periodontally healthy patients	Curette	Culture	*Сandida* spp. were detected in 6 (14.3%) patients with CP 9 (25%) periodontally healthy patients	Included in the meta‐analysis.
12	**Brusca et al., 2014, Argentina** a (Brusca et al., 2014)	Cross‐sectional	Male aged 19−40 years, taking and not‐taking anabolic steroids.	92 subjects: 69 patients with CP 23 periodontally healthy patients	Paper points	Culture	*Сandida* spp. were detected in 51 (73.9%) patients with CP 18 (78,3%) periodontally healthy patients *C. albicans* was detected in: 16 (23.2%) patients with CP 11 (47.8%) periodontally healthy patients *C. dubliniensis* was detected in: 6 (8.7%) patients with CP 2 (8.7%) periodontally healthy patients *C*. *glabrata* was detected in: 7 (10.1%) patients with CP 0 periodontally healthy patients *C. gulliermondii* was detected in: 0 patients with CP 1 (4.3%) periodontally healthy patient	Included in the meta‐analysis.
							*C. krusei* was detected in:	
							5 (7.2%) patients with CP 1 (4.3%) periodontally healthy patient	
							*C. parapsilosis* was detected in:	
							16 (23,2%) patients with CP 2 (8.7%) periodontally healthy patients	
							*C. tropicalis* was detected in:	
							11(15,9%) patients with CP 1 (4.3%) periodontally healthy patient	
13	**Krishnan et al., 2020, India** a (Krishnan et al., 2020)	Cross‐sectional	Male subjects aged 20−50 years	90 subjects 60 patients with CP 30 periodontally healthy patients	Curette	Culture	*Candida* spp were detected in	Included in the meta‐analysis.
							42 (70%) patients with CP 18 (60%) periodontally healthy patients	
							*C. albicans* was detected in	
							21 (35%) patients with CP 13 (43.3%) periodontally healthy patients *C. parapsilosis* was not detected	
							*C. krusei* was detected in	
							11 (18.3%) patients with CP 3 (10%) periodontally healthy patients	
							*C. tropicalis* was detected in	
							8 (13.3%) patients with CP 2 (6.7%) periodontally healthy patients	
							*C. glabrata* was detected in	
							2 (3.3%) patients with CP 0 periodontally healthy patients	
							*Candida* spp density was	
							7785 ± 8070 CFU/ml in patients with CP 4430 ± 4880 CFU/ml in periodontally healthy patients	
14	**Volchenkova et al., 2017, Russia** a (Volchenkova et al., 2017)	Cross‐sectional	Male and female patients aged 20−60 years.	82 subjects: 57 patients with CP 25 periodontally healthy patients,	Paper points	Culture	*Candida* spp. were detected in 37 (64.9%) patients with CP (9.1%) periodontally healthy patients *C. albicans* was detected in 37 (64.9%) patients with CP 2 (9.1%) periodontally healthy patients *C. krusei* was detected in 4 (7.5%) patients with CP 0 periodontally healthy patients *C. glabrata* was detected in 4 (7.5%) patients with CP 0 periodontally healthy patients *C. tropicalis* was detected in 4 (7.5%) patients with CP 0 periodontally healthy patients *C. parapsilosis* was detected in 7 (12.0%) patients with CP 0 periodontally healthy patients *Candida* spp. density was 8.46 ± 0.14 CFU/ml in patients with CP 0.63 ± 0.31 CFU/ml in periodontally healthy patients	Included in the meta‐analysis.
15	**Camargo et al., 2016, Brazil** (Camargo, Abreu, et al., 2016)	Cross‐sectional	Male and female patients aged 27−70 years with CP.	48 patients	Curette	PCR	*Candida* spp. were detected in (the data were taken from the diagrams) 48 (100%) patients with CP *C. albicans* was detected in 28 (58.3%) patients with CP *C. glabrata* was detected in 22 (45.8%) patients with CP *C. tropicalis* was detected in 15 (31.3%) patients with CP *C. dubliniensis* was detected in 48 (100%) patients with CP	No control group (nonsmokers periodontally healthy patients).
16	**Camargo et al., 2016, Brazil** (Camargo, Silva, et al., 2016)	Cross‐sectional	Male and female patients aged 37−62 years with CP.	16 patients with CP	Paper points	PCR	*Candida* spp. were detected in 16 (100%) with CP *C. аlbicans* was detected in 7 (43.8%) patients with CP *C. glabrata* was detected in 7 (43.8%) patients with CP	No control periodontally healthy group.
							*C. tropicalis* was not detected in patients with CP	
							*C. dubliniensis* was detected in	
							16 (100%) patients with CP	
17	**Chumakova et al., 2012, Ukraine** (Chumakova et al., 2012)	Cross‐sectional	Male and female patients aged 18−35 years with mild CP.	56 patients with CP	Curette	Culture	*Candida* spp. were detected in 25 (44.6%) patients with CP *C. albicans* was detected in 25 (46.3%) patients with CP *C. tropicalis* was detected in 1 (2.4%) patient with CP	No control periodontally healthy group.
18	**Tokmakova et al., 2014, Russia** (Tokmakova et al., 2014)	Cross‐sectional	Patients with CP (no data about gender and age)	9 patients with CP	No data	No data	*Candida* spp. were detected in 2 (22%) patients with CP *C*. *albicans* was detected in 1 (11%) patient with CP C. *glabrata* was detected in 1 (11%) patient with CP	No control periodontally healthy group.
19	**Mirsaeva et al., 2018, Russia** (Mirsaeva et al., 2018)	Cross‐sectional	Male and female patients aged 30−44 with CP.	189 patients with CP	Paper points, impression smears	Culture, PCR	Yeast‐like fungi were detected in 79 (41.8%) patients with CP *C. albicans* was detected in 63 (33.3%) patients with CP *C. krusei* was detected in 21 (11.1%) patients with CP *C. stellatoidea* was detected in 3 (1.6%) patients *C. tropicalis* was detected in 2 (1.1%) patients with CP *Candida* spp. density was: 5.74 ± 0.06	No control periodontally healthy group.
20	**Grigoryan et al., 2019, Russia** (Grigoryan et al., 2019)	Cross‐sectional	Male and female patients with CP.	171 patients with CP	Paper points	Real‐time PCR	*Candida* spp. were detected in 39 (22.8%) patients with CP *C. albicans* was detected in 23 (13.5%) patients with CP Other *Candida* spp. were detected in 16 (9.4%) patients with CP	No control periodontally healthy group.
21	**Sasikumar et al., 2017, India** (Sasikumar et al., 2017)	Cross‐sectional	Male and female patients aged 30−55 years with CP.	108 patients with CP	Curette	Culture	*C. albicans* spp. were detected in 20 (18.5%) patients with CP	No control periodontally healthy group.
22	**Joshi et al., 2012, India** (Joshi et al., 2012)	Cross‐sectional	Male and female patients aged 40−60 years with CP.	40 patients with CP	Curette	Culture	*Candida* spp. were detected in 3 (7.5%) patients with CP.	Number of periodontally healthy patients is not indicated.
23	**Risovannaya et al., 2019, Russia** a (Risovannaya & Lalieva, 2019)	Cross‐sectional	Male and female patients aged 35−44 years with CP.	67 subjects: 45 patients with CP 22 periodontally healthy patients	Paper points	Culture	*Candida* spp. were detected in 12 (26.3%) patients with CP 0 periodontally healthy patients *Candida* spp. density was 4.61 ± 0.10 lg CFU/ml in patients with CP 0 lg CFU/ml in periodontally healthy patients	Included in the meta‐analysis.
24	**Komlev et al., 2010, Russia** a (Komleva et al., 2010)	Cross‐sectional	Patients aged 18−71 years with CP (no data about gender and age).	310 subjects 288 patients with CP 22 periodontally healthy patients	No data	Culture	*Candida* spp. were detected in 75 (26.0%) patients with CP 3 (13.6%) periodontally healthy patients *C. albicans* was detected in 51(17.7%) patients with CP 2 (9.1%) periodontally healthy patients	Included in the meta‐analysis.
25	**Melekhov et al., 2013, Russia** a (Melekhov et al., 2013)	Cross‐sectional	Male patients aged 20−50 years.	190 subjects 160 patients with CP 30 periodontally healthy patients	Paper points	Culture	*Candida* spp. were detected in 44 (55%) 0 patients with CP 8 (26.6%) *Candida* spp. density was 1.95 ± 1.37 lg CFU/ml in patients with CP 1.5 ± 0.8 lg CFU/ml in periodontally healthy patients	Included in meta‐analysis
26	**Tsarev et al., 2012, Russia** (Tsarev et al., 2012)	Cross‐sectional	Patients aged 23−69 years with CP	60 patients with moderate CP.		Culture, PCR	*Candida* spp. were detected in 17 patients (28.3%) with CP *C. albicans* was detected in 17 (28.3%) patients with CP *C*. *krusei* was detected in 2 (3.3%) patients with CP *Candida* spp. density was 5.2 ± 0.4 CFU/ml in patients with CP	No control periodontally healthy group.

Abbreviations: CFU, colony‐forming unit; CP, chronic periodontitis, DM, diabetes mellitus.

^a^ The study is included in the meta‐analysis.

The age of the subjects was from 18 to 80 years old. Three studies (Brusca et al., 2014; Krishnan et al., 2020; Melekhov et al., 2013) included only male subjects while subjects from both sexes participated in the other studies. A total of 2717 subjects were examined. Four hundred and sixty‐three of them had a clinically healthy periodontium while 2254 had CP of differing severity. Examination data from patients with systemic pathology were not included. Periodontal pocket or gingival sulcus samples were collected either by a paper point (paper point size and time in the periodontal pocket/gingival sulcus varied) (Brusca et al., 2014; Camargo, Silva, et al., 2016; Canabarro et al., 2013; De‐La‐Torre et al., 2018; Gomes et al., 2017; Grigoryan et al., 2019; Matić Petrović et al., 2015, 2019; McManus et al., 2012; Melekhov et al., 2013; Mirsaeva et al., 2018; Rams et al., 2014; Razina et al., 2017; Risovannaya & Lalieva, 2019; Sanz‐Sánchez et al., 2016; Tsarev et al., 2012; Volchenkova et al., 2017), or a sterile curette (Camargo, Abreu, et al., 2016; Chumakova et al., 2012; Joshi et al., 2012; Krishnan et al., 2020; Matić Petrović et al., 2015, 2019; McManus et al., 2012; Sardi, Almeida, et al., 2011; Sasikumar et al., 2017; Colombo et al., 2016). In three studies (Matić Petrović et al., 2015, 2019; McManus et al., 2012), the samples were obtained by both paper points and sterile curettes. In one study, however (Matić Petrović et al., 2015), all the obtained samples were microbiologically processed together. In two studies, samples were tested separately and the results were then compared (Matic Petrovic et al., 2019; McManus et al., 2012). In two studies (Komleva et al., 2010; Tokmakova et al., 2014), the sample collection technique was not indicated although where the sample was obtained from, that is, the periodontal pocket or gingival sulcus, was indicated. The sample was collected at the deepest periodontal pocket although one study also collected samples at sites without signs of bleeding on probing in patients with CP (McManus et al., 2012). The tooth was isolated from the saliva and the supragingival plaque was removed from the tooth surface by a cotton swab before sample collection. *Candida* spp. were identified using a culture method (Brusca et al., 2014; Canabarro et al., 2013; Chumakova et al., 2012; Joshi et al., 2012; Komleva et al., 2010; Krishnan et al., 2020; Matić Petrović et al., 2015, 2019; Melekhov et al., 2013; Rams et al., 2014; Razina et al., 2017; Risovannaya & Lalieva, 2019; Sanz‐Sánchez et al., 2016; Sasikumar et al., 2017; Volchenkova et al., 2017), PCR (Camargo, Abreu, et al., 2016; Camargo, Silva, et al., 2016; Sardi, Duque, Höfling, et al., 2012), real‐time PCR (Grigoryan et al., 2019), a combination of culture and PCR (De‐La‐Torre et al., 2018; Gomes et al., 2017; McManus et al., 2012; Mirsaeva et al., 2018; Tsarev et al., 2012), or checkerboard DNA−DNA hybridization technique (Colombo et al., 2016). One study did not indicate the identification method (Tokmakova et al., 2014). All studies determined the *Candida* spp. detection rate. Some studies identified the spp.: *C. albicans* was isolated in 16 studies (Brusca et al., 2014; Camargo, Abreu et al., 2016; Camargo, Silva et al., 2016; Chumakova et al., 2012; Grigoryan et al., 2019; Joshi et al., 2012; Komleva et al., 2010; Krishnan et al., 2020; McManus et al., 2012; Mirsaeva et al., 2018; Rams et al., 2014; Sardi, Duque et al., 2011; Sasikumar et al., 2017; Tokmakova et al., 2014; Tsarev at al., 2012; Volchenkova et al., 2017) *С. dubliniensis* in 5 studies (Brusca et al., 2014; Camargo, Abreu, et al., 2016; Camargo, Silva, et al., 2016; McManus et al., 2012; Sardi, Duque, et al., 2011), *C. glabrata* in 8 studies (Brusca et al., 2014; Camargo, Abreu, et al., 2016; Camargo, Silva, et al., 2016; Krishnan et al., 2020; McManus et al., 2012; Sardi, Duque, et al., 2011; Tokmakova et al., 2014; Volchenkova et al., 2017), *C. tropicalis* in 6 studies (Brusca et al., 2014; Camargo, Abreu, et al., 2016; Camargo, Silva, et al., 2016; Krishnan et al., 2020; Mirsaeva et al., 2018; Volchenkova et al., 2017), *C. parapsilosis* in 4 studies (Brusca et al., 2014; Krishnan et al., 2020; McManus et al., 2012; Volchenkova et al., 2017), *C. krusei* in 5 studies (Brusca et al., 2014; Krishnan et al., 2020; Mirsaeva et al., 2018; Tsarev et al., 2012; Volchenkova et al., 2017), *Candida kefyr* (McManus et al., 2012), *Candida stellatoidea* (Mirsaeva et al., 2018) and *Candida gulliermondii* (Brusca et al., 2014) in 1 study. *Candida* spp. density was counted in 10 studies (De‐La‐Torre et al., 2018; Krishnan et al., 2020; McManus et al., 2012; Melekhov et al., 2013; Mirsaeva et al., 2018; Razina et al., 2017; Risovannaya & Lalieva, 2019; Tsarev et al., 2012; Colombo et al., 2016; Volchenkova et al., 2017) *Candida* spp. were not detected in subjects with clinically healthy periodontium in one study (Risovannaya & Lalieva, 2019), while another (Rams et al., 2014) did not detect *Candida* spp. in patients with CP. In other studies, the *Candida* spp. detection rate ranged from 9.1% to 78.3% in patients with clinically healthy periodontium, and from 14.3% to 100% in patients with CP. Regarding the individual spp., the detection rate in patients with clinically healthy periodontium was between 9.1% and 47.8% for *C. albicans*, from 0% to 8.7% for *С. dubliniensis*, from 0% to 2% for *C. glabrata*, from 0% to 6.7% for *C. tropicalis*, from 0% to 8.7% for *C. parapsilosis*, from 0% to 10% for *C*. *krusei*, and 4.3% for *C. gulliermondii*. *C. kefyr* and *C. stellatoidea* were not detected. In CP groups, the *C. albicans* detection rate ranged from 7.5% to 100%, *С. dubliniensis* from 4.8% to 100%, *C*. *glabrata* from 0% to 90%, *C*. *tropicalis* from 0% to 40%, *C*. *parapsilosis* from 4.8% to 23.2%, *C. krusei* from 3.3% to 18.3%, *C. kefyr* from 0% to 4.8%, *C. stellatoidea* was 1.6%, while *C*. *gulliermondii* was not detected.

The heterogeneity of the received data made it necessary to undertake a meta‐analysis. The meta‐analysis involved 11 studies (Brusca et al., 2014; Canabarro et al., 2013; Colombo et al., 2016; Komleva et al., 2010; Krishnan et al., 2020; Matic Petrovic et al., 2015, 2019; Melekhov at al., 2013; Razina et al., 2017; Risovannaya & Lalieva, 2019; Volchenkova et al., 2017) for which the OR of CP development could be calculated if *Candida* spp. were found in the samples taken from the periodontal pockets or crevicular fluid.

### 3.4 Meta‐analysis

#### 3.4.1 Comparison of *Candida* spp. detection rate in patients with clinically healthy periodontium and CP

**Figure 2:**
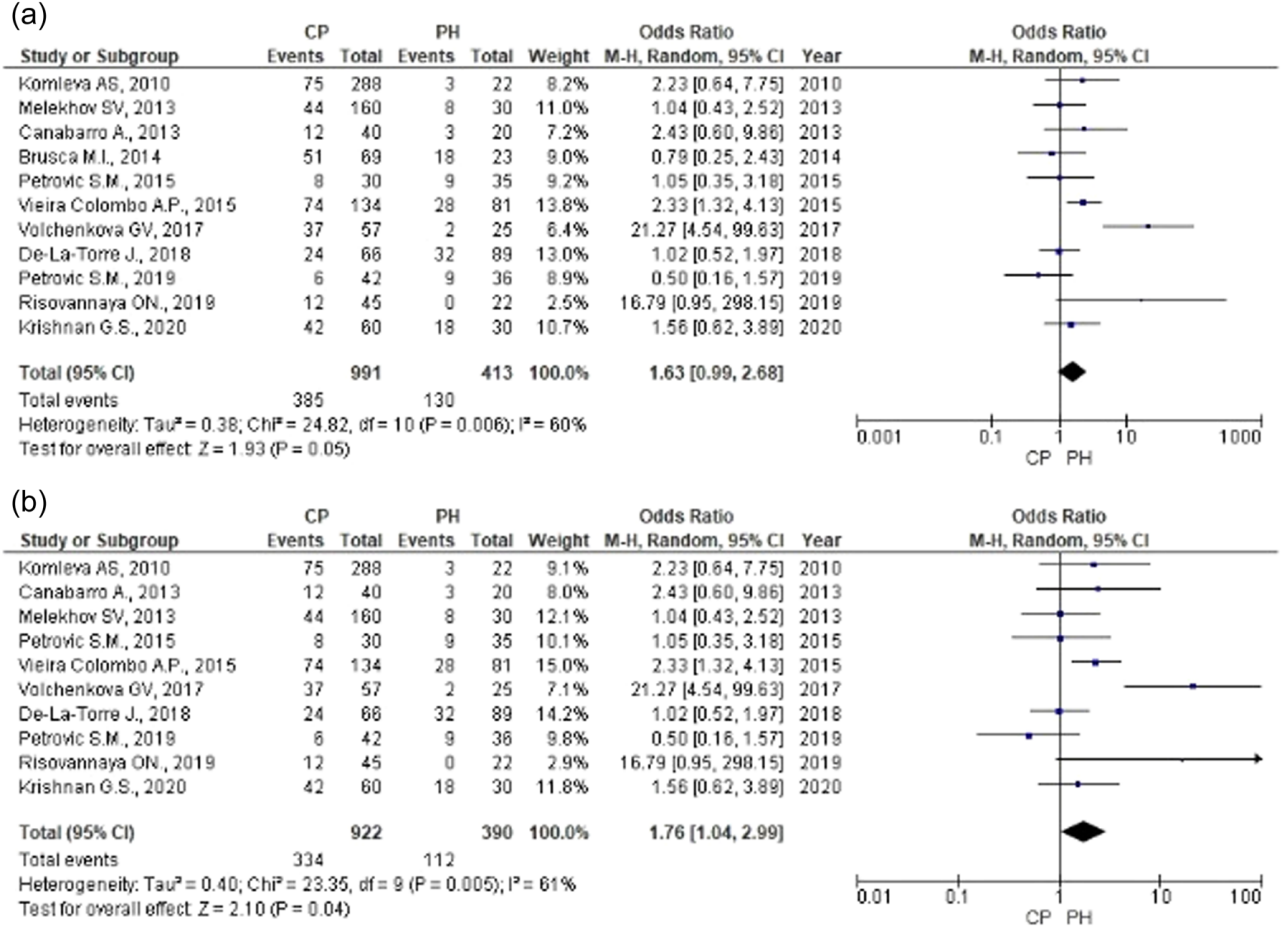
Comparison of *Candida* spp. detection rate. (a) Comparison of *Candida* spp. detection rate in CP patients and periodontally healthy patients. (b) Comparison of *Candida* spp. detection rate in CP patients and periodontally healthy patients (after the exclusion of the study where the control group involved CP patients and periodontally healthy patients). CP, chronic periodontitis; PH, periodontal health.

A total of 1404 subjects were examined, 413 of which did not have any signs of inflammatory periodontal diseases while 991 had CP. If the samples were obtained by both paper points and curettes in the same study, the results were combined. The results demonstrated that *Candida* spp. increased the risk of CP development by 1.63 times but it was not statistically significant (OR = 1.63, 95% CI = 0.99–2.68, *Z* = 1.93; *p* = .05) (Figure 2a). Significant heterogeneity (*χ* ^2^ = 24.82, *p* = .006, *I* ^2^ = 60%) was observed across the studies; the data were analyzed by random effects model. Sensitivity analysis showed that the result was not stable. However, the result differed from the previous conclusion after the exclusion of the study by Brusca et al. (2014) (Figure 2b). Statistically significant data showed that *Candida* spp. could increase the risk of CP development by 1.76 times (OR = 1.76; 95% CI = 1.04–2.99; *Z* = 2.10; *p* = .04). The study by Brusca et al. (2014) included subjects with gingivitis in the control group of subjects with clinically healthy periodontium.

#### 3.4.2 Comparison of the detection rate of individual *Candida* spp.

Based on the results of the previous comparison, we decided to exclude the Brusca et al. (2014) study as a possible heterogeneity source.

The rates of detection of *C. albicans, C. glabrata, C. krusei*, and *C. tropicalis* were compared and their potential role in CP development was determined.

##### 3.4.2.1 C. albicans

**Figure 3:**
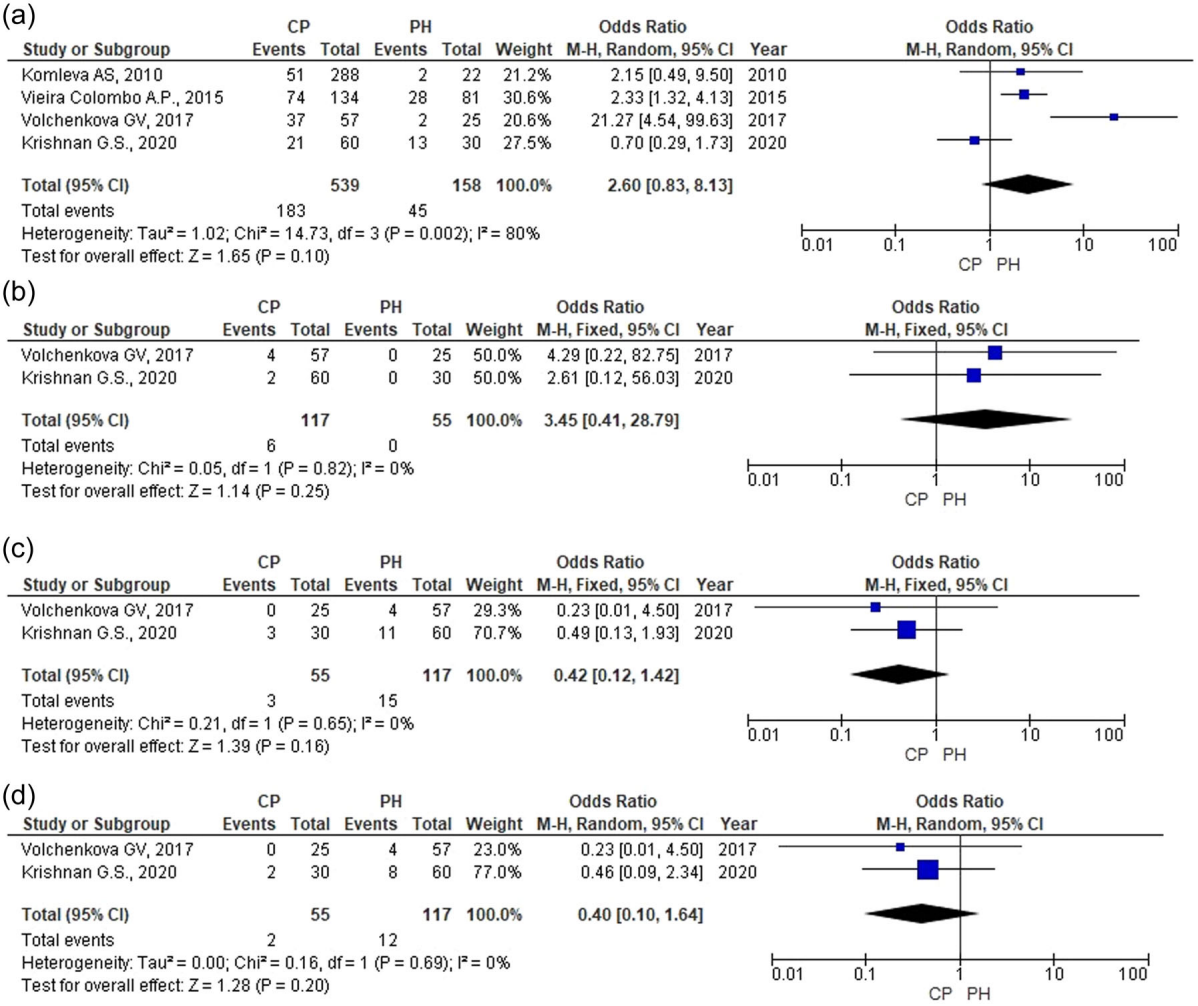
*Candida* spp. detection rate in CP patients and periodontally healthy patients. (a) *Candida albicans*. (b) *Candida glabrata*. (c) *Candida krusei*. (d) *Candida tropicalis*. CP, chronic periodontitis; PH, periodontal health.

No data were received about the statistically significant impact of *C. albicans* on CP development (OR = 2.60; 95% CI = 0.83–8.13; *Z* = 1.65; *p* = .10) (Figure 2). High heterogeneity was observed across studies (*χ* ^2^ = 14.73; *p* = .002; *I* ^2^ = 80%); the data were analyzed by random effects model. Sensitivity analysis demonstrated that the result was not stable. The result differed from the previous conclusion (Figure 3a) after the study by Krishnan et al. (2020) was excluded. There was, however, no grounds to exclude the study from the meta‐analysis.

##### 3.4.2.2 C. glabrata

No data was received about a statistically significant influence of *C. glabrata* on CP development (OR = 3.45; 95% CI = 0.41–28.79; *Z* = 1.14; *p* = .25) (Figure 2). Insignificant heterogeneity was observed across studies (*χ* ^2^ = 0.05; *p* = .82; *I* ^2^ = 0%) while the data were analyzed by fixed effect model (Figure 3b).

##### 3.4.2.3 C. krusei

No data were received about the statistically significant impact of *C. krusei* on CP development (OR = 0.42; 95% CI = 0.12–1.42; *Z* = 1.39; *p* = .16) (Figure 3c). Insignificant heterogeneity was observed across studies (*χ* ^2^ = 0.21; *p* = .65; *I* ^2^ = 0%). The data were analyzed by the fixed effect model.

##### 3.4.2.4 C. tropicalis

No data were received about the statistically significant impact of *C. tropicalis* on CP development (OR = 0.38; 95% CI = 0.09–1.56; *Z* = 1.34; *p* = .18) (Figure 3b). Insignificant heterogeneity was observed across studies (*χ* ^2^ = 0.16; *p* = 0.69; *I* ^2^ = 0%). The data were analyzed by the fixed effect model.

#### 3.4.3 *Candida* spp. density count assessment

**Figure 4:**
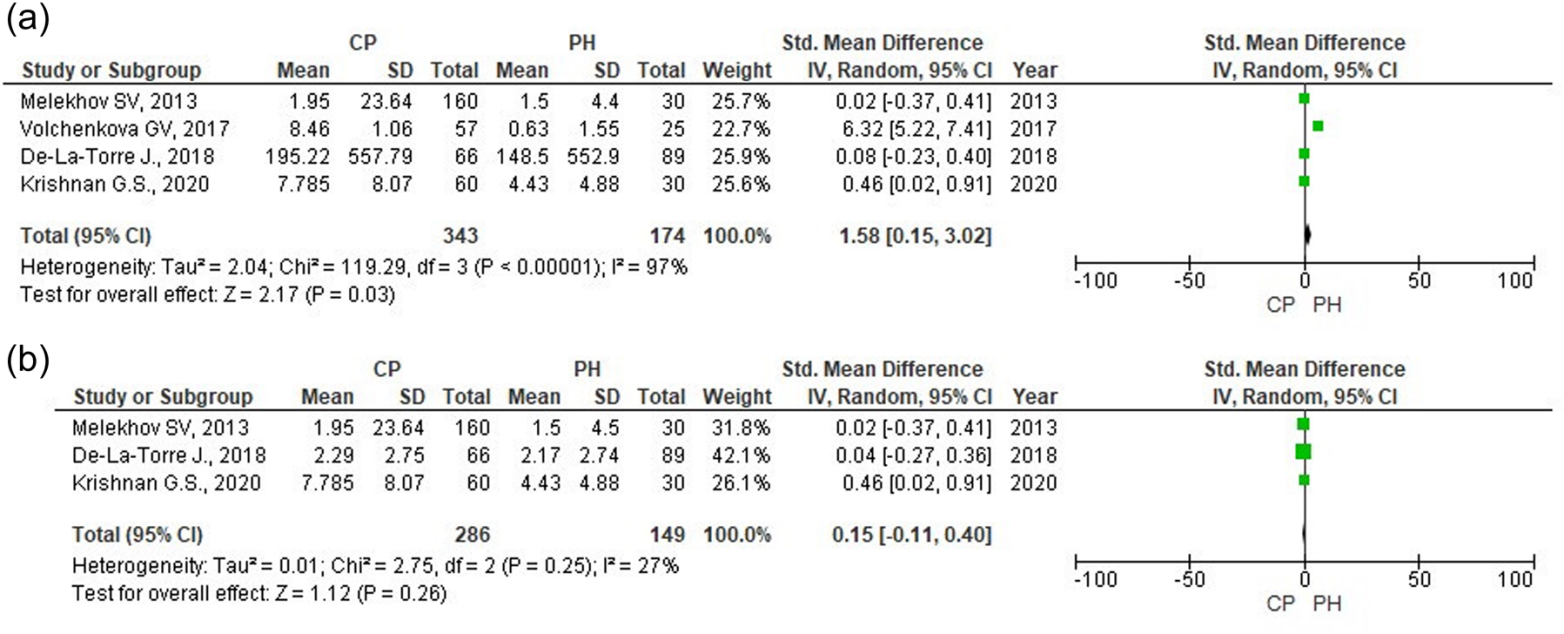
Density of *Candida* spp. (a) Density of *Candida* spp in CP patients and periodontally healthy patients. (b) Density of *Candida* spp in CP patients and periodontally healthy patients (after exclusion of Volchenkova et al. study). CP, chronic periodontitis; PH, periodontal health.

Four studies (De‐La‐Torre et al., 2018; Krishnan et al., 2020; Melekhov et al., 2013; Volchenkova et al., 2017) which contained quantitative data were selected for the comparative assessment of *Candida* spp. density count in subjects with clinically healthy periodontium and CP patients. The SMD was analyzed as the quantitative results had different assessment scales (CFU/ml and lg CFU/ml). The statistically significant data confirmed that more *Candida* spp. were found in periodontal pockets of CP patients than in subjects with clinically healthy periodontium (SMD = 1.58; 95% CI = 0.15‐3.02; *Z* = 2.17; *p* = .03). High heterogeneity was distinguished across studies (*χ* ^2^ = 120.24; *p* ˂ 0.00001; *I* ^2^ = 98%); random effects model was used for data analysis (Figure 4a). Sensitivity analysis proved that the result was not stable. The exclusion of Volchenkova et al. (2017) significantly changed the result; the heterogeneity decreased to insignificant (*χ* ^2^ = 2.75; *p* = .25; *I* ^2^ = 27%), and the overall analysis effect changed, that is, the density of *Candida* spp. was not statistically significantly different (SMD = 0.15; 95% CI = −0.11‐0.40; *Z* = 1.12; *p* = .26) (Figure 4b). However, there were no reasons for the exclusion of the study from the meta‐analysis.

### 3.5 Publication bias analysis

**Figure 5:**
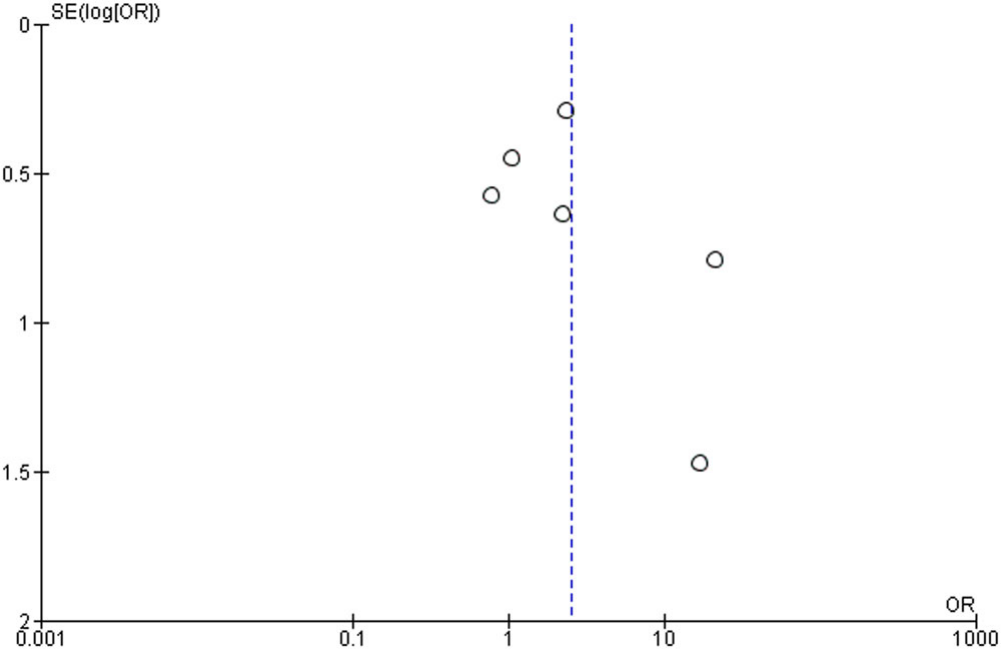
Publication bias analysis. The studies with a small number of patients evidently suggest more frequent *Candida* spp. detection in CP patients and no small study exists to counter this result.

Publication bias was analyzed by a funnel plot (Figure 5).

Asymmetry to the central tendency axis and asymmetry to the axis in the area of larger values of the *y *axis are observed which certifies a publication bias in studies with few subjects.

The studies with a small number of patients evidently suggest more frequent *Candida* spp. detection in CP patients and no small study exists to counter this result. However, the results of the large studies are evenly distributed along the central tendency axis which shows that there is no publication bias in such studies. Hence, it is not possible to unambiguously determine publication bias in the present meta‐analysis.

## 4 DISCUSSION

Bacteria *P. gingivalis*, *T. forsythia, T. denticola*, and *A. actinomycetemcomitans* are pathogens which are most frequently associated with inflammatory periodontal diseases (Socransky et al., 1998). While the evidence of the role of yeast in the development of these diseases is scarce. Though the isolation of *Candida* spp. from the oral cavity per se does not suggest the disease, there are different opinions about the blasts isolated from the periodontal pockets (Matic Petrovic et al., 2019). Opportunistic *C. albicans* prevails among other species of its genus in inflammatory periodontal diseases. Its hyphae have been seen in the periodontal connective tissue and it is associated with highly invasive anaerobic bacteria, such as *P. gingivalis, P. intermedia*, and *A. actinomycetemcomitans* (De‐La‐Torre et al., 2018; Suresh et al., 2020). At the same time, recent studies indicate that *Candida* spp. are commensal microorganisms and belong to normal oral microbiota, colonizing the oral mucosa and tongue and are also found in the supra—and subgingival microbial plaque and saliva in patients without signs of periodontal inflammation or oral candidiasis (Matic Petrovic et al., 2019). Diseases such as HIV, diabetes mellitus, and cancer which cause immunosuppression, as well as certain medications (antibiotics, immunosuppressants, chemotherapy drugs) activate the pathogenic characteristics of *Candida* spp. and increase colonization of the oral habitats (Lomeli‐Martinez et al., 2019; Matic Petrovic et al., 2019; Sardi, Duque, Mariano, et al., 2012; Sun et al., 2016). The prevalence and importance of other *Candida* spp. are of increasing interest, for example, *C. glabrata*, *C. tropicalis*, *C. krusei*, and *C. dubliniensis*, which have recently been isolated from the oral cavity not only in immunocompromised, but also in healthy subjects (Matic Petrovic et al., 2019; Quindós et al., 2018).

Studies report higher carriage of *Candida* spp. in patients with periodontal diseases (Canabarro et al., 2013; Peters et al., 2017). The data published in the literature confirms that *Candida* spp. contribute to the more severe course of CP (Canabarro et al., 2013; Machado et al., 2011). Yeasts and periodontal pathogens can interact physically, chemically, and metabolically to influence microbial survival, colonization, and biofilm formation (Bartnicka et al., 2019; Chevalier et al., 2018; Montelongo‐Jauregui et al., 2016). Anaerobic environment of the periodontal pocket can promote virulences *of Candida* spp. increasing the secretion of proteinases that damage tissues, modulate the immune response, and attract other periodontopathogens (Lafuente‐Ibáñez de Mendoza et al., 2021; Rosa et al., 2008). This contributes to the formation of thick polymicrobial biofilms (Montelongo‐Jauregui & Lopez‐Ribot, 2018; Montelongo‐Jauregui et al., 2019; Young et al., 2020). It is worth mentioning that in the majority of clinical studies, *Candida* spp. were not isolated from the periodontal pockets but from the saliva (Machado et al., 2011; McManus et al., 2012; Peters et al., 2017; Venkatesan et al., 2015), different sites of the oral mucosa (Monroy‐Pérez et al., 2020; Olczak‐Kowalczyk et al., 2015) (cheek, tongue, gingiva) of CP patient. While even saliva isolation and supragingival plaque removal before sample collection are significant for qualitative and quantitative identification of these fungi. Furthermore, the studies were designed without the control group of subjects with clinically healthy periodontium (Chumakova et al., 2012; Gomes et al., 2017; Grigoryan et al., 2019; Mirsaeva et al., 2018; Razina et al., 2017; Sanz‐Sánchez et al., 2016; Sasikumar et al., 2017; Tokmakova et al., 2014; Tsarev et al., 2012).

The present work represents a quantitative analysis, which combines the results of independent studies with different designs. The meta‐analysis of 11 selected studies demonstrated that in CP patient group, the detection rate of *Candida* spp. was statistically significantly higher and increased the risk of CP development by 1.76 times compared to the group of subjects with clinically healthy periodontium. Statistically significant data also confirmed that a higher density of *Candida* spp. was detected in periodontal pockets of CP patients than in subjects with clinically healthy periodontium. However, these data should be interpreted with care as a high heterogeneity across the studies was observed; publication bias was present across the studies where *Candida* spp. were more frequently detected and their (*Candida* spp.) density was higher in advanced periodontitis and the studies had a moderate or high risk of systemic error. There are few studies of species such as *C. glabrata, C. krusei, C. tropicalis*, *C. parapsilosis*. For example, our meta‐analysis detected only two studies that corresponded to the inclusion criteria and that compared the detection rate of *C. glabrata, C. krusei, C. tropicalis* in periodontal pocket samples and crevicular samples in CP patients and subjects with clinically healthy periodontium respectively (Krishnan et al., 2020; Volchenkova et al., 2017). The detection rate of these species was similar in both groups. Colonization of periodontal pockets by these microorganisms did not necessarily certify their activity in the pathogenesis of periodontitis. They can be transient members of the microbial consortium and be evaluated as a potential reservoir for systemic distribution in case of favorable conditions (Matic Petrovic et al., 2019). Thus, an accurate conclusion requires longitudinal but not cross‐sectional studies, as well as studies on the immune response to different spp. and morphological forms of *Candida* spp. (Matic Petrovic et al., 2019).

## 5 CONCLUSIONS

In conclusion, we would like to emphasize that our meta‐analysis is one of the first that critically assesses the detection rate and density counts of *Candida* spp. in CP. The meta‐analysis results demonstrated that *Candida* spp. detection rate and density were statistically significantly higher in CP patients than in subjects with clinically healthy periodontium. Thus, the periodontal pocket may be a niche for the existence of *Candida* spp. Whether this fact is the cause or consequence of periodontal disease remains unclear. However, the high heterogeneity of the studies included in the analysis should be considered. The detection of yeast species does not prove their role in inflammatory lesion formation in the periodontal tissues. It is of primary importance to detect their invasive ability, which is reflected by the activity of hydrolytic enzymes produced by yeast‐like fungi. Additional large‐scale and more standardized experimental and clinical studies are necessary to clarify the role of *Candida* spp. in the origin and development of inflammatory periodontal diseases.

## AUTHOR CONTRIBUTIONS

*Conceptualization*: Ekaterina Slazhneva, Ekaterina Tikhomirova, Victoria Atrushkevich. *Data curation*: Ekaterina Loboda, Victor Tsarev. *Formal analysis*: Victoria Atrushkevich, Liudmila Orekhova. *Investigation*: Ekaterina Tikhomirova, Victor Tsarev. *Methodology*: Ekaterina Slazhneva, Ekaterina Tikhomirova. *Software*: Ekaterina Loboda. *Validation*: Ekaterina Slazhneva, Victor Tsarev, Liudmila Orekhova. *Writing—Original draft*: Ekaterina Slazhneva, Ekaterina Tikhomirova, Ekaterina Loboda. *Writing—Review and editing*:Victoria Atrushkevich, Liudmila Orekhova, Victor Tsarev.

## CONFLICT OF INTEREST

The authors declare no conflict of interest.

## ETHICS STATEMENT

Given that this is a systematic review and does not contain the results of own original research, no ethical approval was required.

## Data Availability

Data are available on request from the authors. The data that support the findings of this study are available from the corresponding author upon reasonable request.
